# Adequate sleep among adolescents is positively associated with health status and health-related behaviors

**DOI:** 10.1186/1471-2458-6-59

**Published:** 2006-03-08

**Authors:** Mei-Yen Chen, Edward K Wang, Yi-Jong Jeng

**Affiliations:** 1 Nursing Department, Chang Gung Institute of Technology, Wen-Hwa 1^st ^Road, Kwei-Shan, Taoyuan, Taiwan; 2 Neurology Department, Tao-Yuan Veteran Hospital, Kwei-Shan, Taoyuan, Taiwan

## Abstract

**Background:**

Amount of sleep is an important indicator of health and well-being in children and adolescents. Adequate sleep (AS: adequate sleep is defined as 6–8 hours per night regularly) is a critical factor in adolescent health and health-related behaviors. The present study was based on a health promotion project previously conducted on adolescents in Tao-Yuan County, Taiwan. The aim was to examine the relationship between AS during schooldays and excessive body weight, frequency of visiting doctors and health-related behaviors among Taiwanese adolescents.

**Methods:**

A cross-sectional study design, categorical and multivariate data analyses were used. The hypotheses investigated were: high frequency of AS is positively associated with lack of obesity and less frequent visits to doctors; and high frequency AS is positively associated with health-related behavior.

**Results:**

A total of 656 boys (53.2%) and girls (46.8%), ranging in age from 13–18 years were studied between January and June 2004. Three hundred and fifty seven subjects (54%) reported that they slept less than the suggested 6–8 hours on schooldays. A significant negative association was found between low sleep and of the following health-related behaviors: (1) life appreciation; (2) taking responsibility for health; (3) adopting healthy diet; (4) effective stress management; (5) regular exercise; and (6) total AHP score. High frequency AS was associated with low frequencies of obesity after potential confounding factors were controlled. Junior high school adolescents reported significantly higher frequencies of AS than high school participants. Gender, family structure, home location and frequency of television watching or computer use were not significantly associated with AS.

**Conclusion:**

These findings support the proposition that AS is associated with good health status and high-frequency adoption of health-related behavior. Furthermore, these findings suggest that inadequate sleep may be a screening indicator for an unhealthy lifestyle and poor health status. The results might be useful for future research into the development of intervention strategies to assist adolescents who are not receiving enough hours of sleep.

## Background

Adequate sleep (AS) is a critical factor for adolescent health and health-related behaviors. However, the relationships between AS and health status, e.g. frequency of doctor visits, non-obesity and health-related behaviors, are not well understood. Humans spend almost a third of their lifetimes sleeping; quality sleep is essential to human health. Sleep is a state of unconsciousness from which one can be aroused. More than a periodic rest condition for the body and nervous system, it is a phase during which the body and nervous system can recuperate. Notably, protein synthesis is more active during sleep than during waking hours [1]. Studies have shown that the average amount of sleep per night for prepubescents, mid-adolescents and old-adolescents was 10, 8 and 7 hours, respectively [2]. Most research has proposed that adolescents require at least 6–8 hours of sleep each night [3,4]. Amount of sleep is an important indicator of health and well-being in children and adolescents. In adolescents, sleep influences physical and emotional well-being (brain maturation, substantial biological and psychosocial changes in puberty, and the interaction between physical and psychosocial domains [5,6]).

Sleep disturbance or deprivation may lead to daytime sleepiness and decreased mental acuity and thus negatively affect normal growth and the ability of adolescents to learn in school [7-9]. Although progress has been made in these areas (growth and learning), there are still major gaps in existing knowledge and a paucity of well-controlled studies to guide specific health policies for adolescent sleep. Some studies have focused on the relationship between healthy sleep habits and daytime sleepiness among children [10,11]. Others have focused on topics related to sleep pattern disturbance during hospitalization [6]. Giannotti et al. [7] investigated the relationship between sleep and motor vehicle accidents. Research specifically addressing the effects of inadequate sleep on obesity/non-obesity, frequency of doctor visits and health-related behavior in adolescents seems scarce.

Primary health-care providers can assist in assessing inadequate sleep in adolescents and educating them about the importance of AS and the consequences of sleep deprivation. Less is known about the role of AS in health-promoting behaviors. Taiwanese adolescents commonly have inadequate sleep (IAS) and the incidence of associated unhealthy behaviors is typically high [12]. According to the study conducted in Taiwan by Soong et al. [12], junior high adolescents received an average of less than 7 hours (418.35 ± 68.84 minutes) of daily sleep when school was in session. Unfortunately, the unhealthy behaviors related to IAS are not a priority for health services in Taiwan. The seriousness of issues related to IAS in adolescents is not well recognized and is usually under-reported in Taiwan [13]. For example, there was no reference to sleep issues for adolescents in the 13 health goals published by the Taiwanese Division of Child and Adolescent Health. In the light of this omission, the present study examined the relationship between AS, health status and health-related behaviors.

## Methods

### Study design and population

This study is part of a 3-year health promotion project comparing the differences in health-related behavior between overweight and non-overweight adolescents in Tao-Yuan County, Taiwan. A cross-sectional descriptive design was used. There are 51 public junior-high and high schools in Tao-Yuan County. To enroll a representative study group, participants were recruited from seven selected schools located in rural (three schools) and urban (four schools) areas that were, respectively, more than 30 km and less than 3 km distant from downtown Tao-Yuan city.

To balance the sample size and gender distribution between overweight and non-overweight samples in the original project, the researchers evaluated BMI data from the school health center before sending the invitation letter. Study subjects comprised nearly half overweight and half non-overweight students aged 13–18 years in grades 7–11 during the first trimester of the school year. They were selected according to the following inclusion criteria: (a) body mass index (BMI), recorded at the respective school health center, was in the rage of 15–30; (b) both the student and the student's guardian provided written consent; (c) there was no physical or mental limitation.

### Human subject protection and data collection procedures

The study was approved by the Institutional Review Board (CGMH, No 2004–6145). Informed written consent was obtained from the subjects and their guardian(s) after permission had been obtained from the relevant school administrators. Parents were fully informed about the survey and had an opportunity to review the questionnaire; that is, an invitation letter, emphasizing that the responses would be confidential, were sent together with the questionnaire to each student's guardian. The investigators explained the study to all the students. Students completed the questionnaire anonymously and used about 30 minutes to fill out the scale. They could decline to participate in the project at any time while completing the questionnaire.

### Measurements and statistical analysis

**Demographic characteristics **were as follows: Gender; age; family's religion; home location (urban/rural); parents' educational level; and family structure (whether students lived with both parents, a single mother/father, grandparents, or other relatives). Television watching and computer use: two variables were measured, (1) mean hours of TV watching or computer use from Monday through Friday, (2) mean hours of TV watching/computer use during weekends and holidays.

For time of usage of TV/computer on weekend and weekdays, we defined computer usage time as "Excluding the school's requirements (e.g. get information for doing homework), I usually used computer for playing games or chatting with someone", while watching TV was defined as "the time during which the TV was turned on and off on weekdays".

Adequate sleep (AS) was defined as 6–8 hours of sleep per night on more than four weekdays per week (this definition was drawn from the literature and real situations). Inadequate sleep (IAS) was defined as 6–8 hours per night on fewer than three weekdays. To obtain valid reporting of sleeping time per night and to ensure the reliability and validity of the measurement, the method we used was established in three stages. First, three junior-high and two high school adolescents were invited to participate in a pilot study that was a test of content validity. None of the students appeared to have any doubt about their own measurement of their sleeping times. Next, all participants were given instructions on counting their sleeping hours each night during the study period: for example, "if you go to bed at 11 PM and awaken at 6 AM, then you obtain total 7 hours of sleeping time". Finally, the students responded to a self-administered questionnaire with a Likert scale: "generally speaking, I sleep at least 6–8 hours each night", self-rating the frequency as one choice among "rarely, sometimes, usually, always". These frequencies were defined below the question: for example, "rarely" represented "I have 6–8 hours per weekday night on fewer than 2 nights"; "sometimes" represented "about three nights"; "usually" represented "4 nights"; and "always" represented "5 nights".

*Health status *was determined by two factors: (1) Body Mass Index (BMI), and (2) frequency of health insurance clams. Height and weight were measured when participants were dressed in light indoor clothing and without footwear. Height was measured to the nearest 0.1 cm, and weight to the nearest 0.1 kg. Each student's BMI was then calculated using the standard formula (weight (in kg) divided by height (in m^2^)). BMI was plotted on the age and sex-specific cutoff points to define the different body sizes of adolescents according to nationally accepted guidelines [14]. Each student was classified as overweight (> 85th percentile for age and sex) or not overweight (> 5th percentile and < 85th percentile). For example, in Taiwan, a 15-year-old boy with a BMI > 23.1 was defined as overweight, whereas a 15-year-old girl with a BMI > 22.7 was considered overweight. Participants also reported how many times had they visited a doctor or been to a hospital for health problems during the past year, and explained the reason for their doctor visit.

*Health-related behaviors *were measured using the Adolescent Health Promotion (AHP) scale [15], which is considered valid and reliable. The AHP comprises 40 items assessing six dimensions of behavior: (1) nutritional (eating breakfast daily, eating 3 meals a day, drinking at least 1,500 cc of water daily, choosing foods with little oil, etc.); (2) social support (speaking to and sharing feelings with others, talking about personal problems with others, keeping in touch with relatives, etc.); (3) life-appreciation (making an effort to feel happy and content, degree of positive thinking, recognition of personal strengths and weaknesses and their acceptance, etc.); (4) health responsibility (reading food labels when shopping, washing hands before meals, standing or sitting up straight, etc.); (5) stress-management (smiling or laughing every day, making schedules, setting priorities, etc.); and (6) exercise behavior (exercising rigorously for 30 minutes at least 3 times per week, performing stretching exercises daily, etc.). The measuring instrument used to obtain the frequency of reported behaviors was a self-reporting Likert scale with a five-point response format: "never, rarely, sometimes, usually, always", with the rating score ranging from 1 to 5. The validity and reliability of the original AHP were assessed with a sample of 1128 Taiwanese adolescents and deemed to be satisfactory. Factor analysis yielded a six-factor instrument that explained 51.14% of the variance in the 40 items. Cronbach's alpha reliability coefficients were 0.93 for the total scale, and the alpha coefficients for the subscales ranged from 0.75 to 0.88 [15].

### Data analysis

SPSS (Version 11.0) was used for all the data analyses. All tests were 2-sided and p-values less than 0.05 were considered statistically significant. Categorical data analyses (e.g. chi-square test, odds ratios and 95% confidence intervals) and multivariate analyses were applied to determine the relationships among AS, health status, health-related behaviors and their associated factors. Before any categorical data analysis, the 6 dimensions of the health-promoting behavior scale were classified as low frequency (if the value was below the mean score of each sub-scale) or high frequency (if the value was above the mean score of each sub-scale).

## Results

### Description of the sample

Of the 890 participants, 179 were excluded from the study owing to the absence of parental consent, and another 55 were removed for the following reasons: 44 had multiple invalid responses, and 11 cited either fatigue or personal reasons for declining to participate. The valid response rate was 73.7%. The study sample therefore comprised 656 boys (53.2%) and girls (46.8%) aged 13–18 years (15.0 ± 1.7). Roughly 69% of the respondents were in middle school (grades 7–9) and 31% in high school. Most fathers and mothers of the participants, 76.8% and 85.3% respectively, had completed middle or high school education. A total of 88% of the subjects lived with both parents. The remaining 12% lived with a single parent or relatives such as grandparents, aunts or uncles. Approximately 34% of the subjects reported no particular religious family background and 37% cited Buddhism or a Buddhism-related religion as the family religion. The mean time of TV watching during weekdays was 2.3 ± 1.4 hours (range, 1–7 hours). The mean time of computer usage during weekdays was 1.6 ± 1.5 hours (range, 0–8 hours). The mean frequency of health insurance claims during the past year was 3.3 (range, 0–50). The predominant self-reported reason for visiting hospitals or doctors was the common cold (n = 340, 51.5%).

Generally, adolescents with frequent AS practiced health-related behaviors (32 items out of the 40) more often than those with infrequent AS. Categorical data analyses show that, with the exception of the social support dimension, adolescents with frequent AS had significantly higher frequencies of healthy behaviors in life appreciation (odds ratio = 1.98, 95% CI = 1.4~2.7), health responsibility (odds ratio = 1.61, 95% CI = 1.2~2.2), stress management (odds ratio = 7.56, 95% CI = 5.3~10.8), nutrition (odds ratio = 2.99, 95% CI = 2.2~4.1) and exercise (odds ratio = 2.15, 95% CI = 1.6~3.0), and a higher overall score for health-promoting behaviors (odds ratio = 2.91, 95%, CI = 2.1~4.0), than students with infrequent AS (Table 1).

Table 2 shows that high AS frequency was strongly correlated with non-obesity (OR = 1.74, 95% CI = 1.3~2.4) and a lower incidence of doctor or hospital visits (OR = 1.71, 95% CI = 1.2~2.4). The junior high students had higher AS frequencies than the high school students (OR = 3.40, 95% CI = 2.4~4.9) (Table 3). Overall, 357 students (54%) reported a low frequency of the recommended 6–8 hours of sleep during school days. However, gender, parental educational levels, living with or without both parents, family religion, hours of watching TV/using computer during weekdays and living in an urban or rural area were not significantly correlated with AS.

Furthermore, when gender and grade factors were excluded, multiple regression analysis showed that the adolescents who achieved a higher frequency of AS also had a significantly higher frequency of practicing health-related behavior in life appreciation (p < 0.001), health responsibility (p < 0.01), stress management (p < 0.001), nutrition (p < 0.001) and exercise (p < 0.001), and a higher total score of adolescent health promotion behaviors (p < 0.001; Table 4). From Table 4 it also appears that, when other factors were excluded, girls had significantly higher scores for healthy behavior in social support (p < 0.001) and higher total AHP scores (p < 0.05) than boys, but lower scores for exercise behavior (p < 0.05). In addition, controlling for gender and grade, achieving frequent AS was negatively associated with obesity (p < 0.001). When gender and grade were excluded, there was no statistically significant association between sleeping time and the frequency of visiting a doctor.

## Discussion

Three key findings emerged from this study. First, AS is positively correlated with the frequency of health-promoting behaviors. Second, AS is positively correlated with a high probability of not becoming overweight and a low frequency of visiting medical doctors. Finally, middle school adolescents had a higher frequency of AS than high school adolescents.

### Adequate sleep is positively associated with health promoting behavior

After the potentially confounding factors of gender and age (grade) were controlled, adolescents who obtained AS had a higher frequency of health-promoting behaviors such as stress management, healthy diet, life appreciation, health responsibility and exercise, and a higher total score of adolescent health promotion behavior, than those who did not obtain AS (Tables 1 and 4). Some of these findings accord with previous studies [3,10,16]. For example, Tynjala et al. [16] found in a Finnish study that good health promotion habits and infrequent use of addictive substances were critical for subjective sleep quality. Self-reported sleep quality was associated with life satisfaction, increased vigor, and decreased fatigue and confusion in adults 40 to 70 years of age. Physically activity was also significantly correlated with sleep quality [3]. Manni et al. [10] found that chronic poor sleep was significantly associated with emotional problems.

Some studies have shown that poor sleep quality is associated with social factors such as difficulty in dealing with problems, increased anxiety and tension, and behavioral problems, and has a negative effect on academic performance [5,17,18]. This study did not investigate the relationship between IAS and academic performance. However, it revealed a relationship between IAS and a negative effect on stress management, such as less smiling or laughing, sharing feelings with others, or making schedules and setting priorities.

### Adequate sleep is positively associated with health status

The findings indicated that adolescents who frequently obtained 6–8 hours of sleep each night had a reduced probability of becoming overweight, even when the potential confounders of gender and grade were excluded (Tables 1 and 4). About 54% of participants reported that they rarely had 6–8 hours of sleep per night on school days; that is, the prevalence of IAS was relatively high. Some studies have shown that sleep deprivation impairs physical functioning and emotional well being [6,16]. Sleep deprivation may also result in lapses of attention, indifference, loss of empathy and reduced motivation [3]. The short-term effects of sleep deprivation in school-age children appear to be manifested as daytime fatigue only; medium-term effects have been associated with daytime sleepiness and behavior problems [2]. Kahn et al. [2] also found that young adults with IAS had significantly reduced lymphocyte responses to chemotactic factors. The present findings show that the dominant reason for visiting doctors was the common cold (n = 340, 51.5%). Although the potential confounding factors of gender and grade were excluded, no association between frequency of AS and number of visits to a doctor was found by multivariate analysis. Another finding revealed that high school adolescents visited a doctor more frequently than did junior high school adolescents (Table 4). This phenomenon deserves to be explored in a future study.

Analytical findings showed that adolescents with low frequencies of AS had an increased probability of being overweight. Sekine et al. [19] obtained similar results during a survey study in Japan and found a significant relationship between reduced amounts of sleep and child obesity. Furthermore, Vioque et al. [20] found that obese people in Spain spent less time sleeping than non-obese. A possible explanation could be a relationship between reduced hours of sleep and increased sympathetic activity, elevated cortisone secretion and decreased glucose intolerance [21]. However, less evidence was available to support this relationship between insufficient sleep and excess weight.

### Why do Taiwanese adolescents obtain less sleep?

Overall, 54% of middle school adolescents reported that they slept less than the suggested 6–8 hours on school days; 74% of high school students reported IAS (Table 3). Comparison of these data with those in other sleep studies revealed that Taiwanese adolescents slept as little as, or less than, those in other countries. For example, Yamaguchi et al. [22] reported that most junior high adolescents in Japan received 6–8 hours of sleep on weekdays. Masalan et al. [23] found that 44.2% of high school adolescents in Chile had sleeping disorders that were associated with environmental conditions (e.g. excessive room temperature) and behaviors (e.g., watching TV before sleep). Van Den [24] revealed that in Belgium, watching T.V. and playing computer games or surfing the Internet were negatively associated with amounts of sleep. In this study, hours spent watching TV or using a computer before going to bed were not significant influences on obtaining AS. This finding is similar to those of Soong et al. [12], that the average nightly sleep obtained by Taiwanese junior high adolescents is less than 7 hours on school days. Fortunately, most of the adolescents in Taiwan also take a nap after lunch for 30–60 minutes in the classroom. Moreover, many adolescents obtained more than 9 hours of sleep per day during weekends [12].

Why then do Taiwanese adolescents obtain less sleep? This might be explained by the fact that in junior high and high school, adolescents are required to spend considerable time completing school assignments and studying for university entrance exams. This probably reflects differences in educational systems among countries. In Taiwan, compulsory fundamental education currently comprises six years of elementary school and three years of junior high education. Upon completion of these nine years, nearly all students must take a national entrance examination that determines whether they enter an academic track or a vocational track high school [25]. The Taiwan government is actively planning to extend compulsory education to cover senior high schooling, and to create a more complete and contemporary educational structure. In Taiwan, as elsewhere in the world, how to enhance lifelong health promotion throughout society, beginning with the youth, is a key issue for the government. However, education reform policies and their implementation need long-term evolution, periodic measurement of outcomes, and adjustment. We hope that the present study will be a useful contribution to that process. Other possible reasons might be that families do not regulate their children's sleep habits and that adolescents are not well skilled at time management. Therefore, further studies should attempt to educate adolescents, parents, teachers and schools about the importance of sleep.

### Limitations of this study

This study has some limitations. First, since the original aim was to explore healthy behavior among overweight and non-overweight adolescents, selection bias was apparent, as nearly half the subjects were overweight. In addition, most of the subjects who were not included in the analysis were excluded owing to the absence of parental consent forms. From brief discussions with those who did not return consent forms, it appeared that Taiwanese students may have been reluctant to present the forms to their parents or guardians, or that the parents were too busy to fill them out. Some of the students living in rural areas said "My parents said yes, you can participate in their study, but did not provide a signature because that looks too formal..." This also constituted a potential selection bias. A second significant limitation of this research was that the data were obtained from only one county in Taiwan, using self-reports rather than random sampling. Third, this study used subjective self-reporting of sleeping hours to determine adequate or inadequate sleep time, and this might have caused significant measurement errors. Further research should use more objective devices such as actigraphy to measure the sleep time; this might enable researchers to measure the quality of sleep hours in individuals. Furthermore, this study did not consider self-perceived sleep quality, factors of sleep disturbance or home atmosphere, although these are important factors for a good sleep.

## Conclusion

This study investigated adolescent sleeping habits in order to identify key factors related to health-promoting behaviors. Knowledge of factors that impact on adolescent sleep behavior is essential for health professionals who want to be responsive to the developmental and lifestyle factors that influence the health of youth. Most studies are concerned with predictors of sleep deprivation and its negative consequences for adolescents, such as incidence of drug abuse or poor school performance. The findings of this research may be valuable in regard to the relationships between IAS and negative health consequences and specific health-promoting behaviors in adolescents. The results further suggest that primary health-care providers should attempt to identify factors that contribute to adolescents not obtaining adequate sleep regularly. To provide a health promotion program that assists adolescents, schools and families requires extra education and support. Interventions that might prove most helpful are parent education classes and discussion groups, from which accurate information regarding sleep issues can be obtained. To improve adolescent health, a change from a school-based approach to an acceptance of personal and corporate responsibility for more healthy lifestyles is necessary. This study's findings could be important for health strategies related to reducing the prevalence of adolescents with IAS and promoting a healthy lifestyle for our youth.

## Competing interests

The author(s) declare that they have no competing interests.

## Authors' contributions

MC: Study design, data collection, data analysis, writing the manuscript

EW: Study design, co-analyzer, participating in writing the manuscript

YJ: Data analysis

## Pre-publication history

The pre-publication history for this paper can be accessed here:



## Figures and Tables

**Table 1:** Relationship between the frequency of AS and health-promoting behavior among the 656 study participants

Variables	Adequate sleep^3^		χ^2 ^(d.f. = 1)	Odds ratio	95% Confidence Interval
	Higher	Lower			
	n %				

Life appreciation					
Higher^1^	202 (52.5%)	183 (47.5%)	17.99***	1.98	1.4~2.7
Lower^2^	97 (35.8%)	174 (64.2%)			
Health responsibility					
Higher	198 (50.1%)	197 (49.5%)	8.65**	1.61	1.2~2.2
Lower	100 (38.3%)	161 (61.7%)			
Social support					
Higher	166 (47.6%)	183 (52.4%)	1.19	1.19	0.9~1.6
Lower	133 (43.3%)	174 (56.7%)			
Stress management					
Higher	230 (67.2%)	110 (32.4%)	145.28***	7.56	5.3~10.8
Lower	68 (21.5%)	248 (78.5%)			
Nutrition					
Higher	180 (60.0%)	120 (40.0%)	46.81***	2.99	2.2~4.1
Lower	119 (33.4%)	237 (66.6%)			
Exercise					
Higher	151 (56.8%)	115 (43.2%)	22.63***	2.15	1.6~3.0
Lower	148 (37.9%)	242 (62.1%)			
Total AHP					
Higher	191 (58.5%)	135 (41.5%)	44.48***	2.91	2.1~4.0
Lower	107 (32.4%)	223 (67.6%)			

^12 ^Higher/lower is defined as the frequency of practicing behavior above/below the average of the total sample scores on the six dimensions of the health promotion scale.

^3^Adequate sleep: Lower/higher was defined as the frequency of obtaining AS (6–8 hours each night); "lower" for rarely/sometimes, and "higher" for "often/always".

** Fisher's exactly test: p < 0.01; *** p < 0.001

**Table 2:** Relationship between frequency of AS and health status indicators

Variables	Adequate sleep		χ^2 ^(d.f. = 1)	Odds ratio	95% Confidence Interval
	Higher	Lower			
	n (%)				
Body size					
Non-overweight	184 (51.7%)	172 (48.3%)	12.11***	1.74	1.3~2.4
Overweight	114 (38.1%)	186 (61.9%)			
Visiting doctors^1^					
Lower	224 (49.7%)	227 (50.3%)	9.82**	1.71	1.2~2.4
Higher	75 (36.6%)	130 (63.4%)			

^1^"Visiting doctors" is defined as frequency of using health insurance.

"Lower" is defined as a frequency of using health insurance below the total sample average (less than or equal to 2×);

"Higher" is defined as a frequency of using health insurance above the total sample average (above or equal to 3×).

** Fisher's exactly test: p < 0.01; *** p < 0.001

**Table 3:** Relationship between frequency of AS and associated factors

Variables	Adequate sleep		χ^2 ^(d.f. = 1)	Odds ratio	95% Confidence Interval
	Higher	Lower			
	n (%)				

Grader					
Middle school (7^th^-9^th^)	246 (54.4%)	206 (45.6%)	47.49***	3.40	2.4~4.9
High school (10^th^-12^th^)	53 (26.0%)	151 (74.0%)			
Gender					
Boys	159 (45.8%)	188 (54.2%)	0.02	1.02	0.7~1.4
Girls	140 (45.3%)	169 (54.7%)			
Family structure					
With both parents	264 (45.7%)	314 (54.3%)	0.02	1.03	0.6~1.7
Others	35 (44.9%)	43 (55.1%)			
Family religion					
Yes	196 (45.2)	238 (54.8)	0.09	1.05	0.8~1.5
None	103 (46.4)	119 (53.6)			
Living location					
Rural	207 (45.6%)	247 (54.4%)	0.00	1.02	0.7~1.4
Urban	92 (45.5%)	110 (54.5%)			
Watching TV (weekday)^1^					
Lower	230 (44.3)	289 (55.7)	1.60	0.78	0.5~1.1
Higher	69 (50.4)	68 (49.6)			
Using Computer (weekday)^2^					
Lower	221 (45.2)	268 (54.8)	0.10	0.90	0.6~1.3
Higher	78 (46.7)	89 (53.3)			

^12^Lower/higher frequency: watching TV or using computer is defined as below/above the average total hours on weekdays for the sample.

**Table 4:** Multiple regression analysis of health-promoting behavior on AS, gender and grade

Variables	LA^1^	HR	SS	SM	NU	EX	TAHP	Body size (OW = 1)^4^	Visiting Dr. (Higher = 1)^5^
Adequate sleep	0.17***^2^	0.11**	0.07	0.47***	0.27***	0.17***	0.27***	-0.16***	-0.07
(Higher)	(0.04)^3^	(0.04)	(0.04)	(0.04)	(0.04)	(0.04)	(0.04)	(0.04)	(0.04)
Gender	0.05	0.07	0.24***	0.01	-0.05	-0.07*	0.09*	-0.12**	0.06
(Girls)	(0.04)	(0.04)	(0.04)	(0.04)	(0.04)	(0.04)	(0.04)	(0.04)	(0.04)
Grader	0.02	-0.00	0.10	0.04	0.03	-0.06	0.03	-0.10*	0.18***
(High school)	(0.04)	(0.04)	(0.04)	(0.04)	(0.04)	(0.04)	(0.04)	(0.04)	(0.04)
Constant	0.48***	0.52***	0.36***	0.29***	0.34***	0.38***	0.33***	0.62***	0.26***
	(0.04)	(0.04)	(0.04)	(0.03)	(0.04)	(0.04)	(0.04)	(0.04)	(0.03)
F-value of ANOVA	6.57***	3.87**	16.55***	58.38***	16.93***	10.31***	17.51***	9.98***	11.61***
R^2^	0.03	0.02	0.08	0.21	0.07	0.05	0.08	0.04	0.05

^1^LA: life appreciation; HR: health responsibility; SS: social support; SM: stress management; NU: nutrition; EX: exercise; TAHP: Total adolescent health promotion score.

^2^Regression coefficient. *** p < 0.001, ** p < 0.01, * p < 0.05.

^3^Standard deviation of regression coefficient.

^4^OW: overweight = 1; non-overweight = 0.

^5^Visiting doctor: higher frequency = 1; lower frequency = 0.
